# Case Report: Dynamics of Acquired Fluoroquinolone Resistance under Standardized Short-Course Treatment of Multidrug-Resistant Tuberculosis

**DOI:** 10.4269/ajtmh.20-0201

**Published:** 2020-07-06

**Authors:** Jean Claude Semuto Ngabonziza, Armand Van Deun, Patrick Migambi, Esdras Belamo Niyigena, Théogène Dusabe, Yves Mucyo Habimana, Bertin Ushizimpumu, Wim Mulders, Tom Decroo, Dissou Affolabi, Philip Supply, Bouke C. de Jong, Leen Rigouts

**Affiliations:** 1 National Reference Laboratory Division, Department of Biomedical Services, Rwanda Biomedical Centre, Kigali, Rwanda;; 2 Mycobacteriology Unit, Department of Biomedical Sciences, Institute of Tropical Medicine, Antwerp, Belgium;; 3 Department of Biomedical Sciences, University of Antwerp, Antwerp, Belgium;; 4 Independent TB Consultant, Leuven, Belgium;; 5 Tuberculosis and Other Respiratory Diseases Division, Institute of HIV/AIDS Disease Prevention and Control, Rwanda Biomedical Centre, Kigali, Rwanda;; 6 MDR-TB Clinic, Kabutare Hospital, Huye, Rwanda;; 7 Department of Clinical Sciences, Institute of Tropical Medicine, Antwerp, Belgium;; 8 Research Foundation Flanders, Brussels, Belgium;; 9 Laboratoire de Référence des Mycobactéries, Cotonou, Benin;; 10 Univ. Lille, CNRS, Inserm, CHU Lille, Institut Pasteur de Lille, U1019-UMR9017-CIIL-Centre d’Infection et d’Immunité de Lille, F-59000 Lille, France

## Abstract

We report a case of acquired fluoroquinolone (FQ) resistance under short-course multidrug-resistant tuberculosis (MDR-TB) treatment. The patient was managed at Kabutare hospital, one of the two specialized MDR-TB clinics in Rwanda. A low dose of moxifloxacin was used in the first three critical months. Acquired resistance was identified at the ninth month of treatment, 3 months after stopping kanamycin in a strain initially susceptible only to FQs, kanamycin, and clofazimine. Fluoroquinolone resistance was detected in the same month by deep sequencing as routinely used second-line line probe assay and phenotypic drug susceptibility testing. High-dose FQ, preferably gatifloxacin, should be used to maximize effectiveness.

## INTRODUCTION

Multidrug-resistant tuberculosis (MDR-TB), defined as TB resistant to at least rifampicin and isoniazid, is among the top global challenges in TB control and management. The two standardized WHO-endorsed MDR-TB treatment regimens, that is, a 9-month short treatment regimen (STR) and a 20-month regimen, include a later generation fluoroquinolone (FQ) as core drug. The core drug of a regimen is the drug that contributes most to achieving relapse-free cure, whereas companion drugs prevent acquisition of resistance to the core drug, and thus prevent treatment failure.^1^

Fluoroquinolone-resistant MDR-TB in Rwanda is very rare; no cases were identified in the 2015 drug resistance survey^2^ and none during routine testing of more than 400 rifampicin-resistant patients (in place since 2010, National Reference Laboratory Rwanda).^3^ Development of TB drug resistance may be induced by bacterial exposure to low drug levels, as a result of either inappropriately low dosing or suboptimal bioavailability of the drug, yet mainly through unknown resistance profiles to companion drugs, effectively resulting in monotherapy.^4,5^

Here, we discuss sequential events preceding the development of FQ resistance in an MDR-TB patient treated with the STR in Rwanda, using targeted deep sequencing (Deeplex-MycTB, GenoScreen, France), with a minimum filtering set at 3% minority reads, alongside second-line LPA (GenoType MTBDR*sl* [Hain Lifescience, Germany]) and phenotypic drug-susceptibility testing (pDST) on subsequent samples to identify the time point at which acquired FQ resistance became detectable.

## CASE REPORT

A 63-year-old, HIV-coinfected woman with no history of TB, diagnosed with rifampicin-resistant TB by Xpert MTB/RIF (Cepheid, Sunnyvale, CA), was referred to Kabutare hospital, one of the two MDR-TB–specialized clinics in Rwanda. Clinically, the patient presented with severe symptoms including fever, severe cough, asthenia, thoracic pain, oxygen desaturation (requiring oxygen therapy), weight loss (37 kg; body mass index = 15.4), epigastric pain, and buccal candidiasis. The patient had been taking antiretroviral treatment since 2005, with 512 CD4 cells/µL and an undetectable viral load at the start of TB treatment. She was started on the STR 1 day after the diagnosis of rifampicin-resistant TB, as per routine practice in Rwanda. As per national guidelines adapted from a previous survey protocol,^6^ based on the patient’s weight, the initiated STR comprised moxifloxacin (200 mg), kanamycin (500 mg), protionamide (500 mg), ethambutol (600 mg), clofazimine (100 mg), isoniazid (300 mg), and pyrazinamide (800 mg).

The second-line LPA, performed on a baseline sample, showed the strain to be susceptible to FQs and second-line injectables. Phenotypic drug-susceptibility testing on the Löwenstein–Jensen (LJ) media confirmed resistance to rifampicin (40 µg/mL), isoniazid (0.2 µg/mL), ethambutol (2 µg/mL), and ethionamide (40 µg/mL), and showed susceptibility to ofloxacin (2 µg/mL) and kanamycin (30 µg/mL). The patient was enrolled in the MDR-TB diagnostic trial “DIAgnostics for MDR-TB in Africa” (DIAMA; Clinicaltrials.gov, NCT03303963). The trial evaluates Deeplex-MycTB, a novel multiplex deep sequencing–based drug resistance diagnostic platform that simultaneously provides sequence information of 18 genes associated with resistance to TB drugs, and new strategies for treatment follow-up. These include fluorescein diacetate (FDA), a vital stain microscopy allowing the distinction between live and dead bacilli.^7^ Routine monitoring included light-emitting diode-fluorescence microscopy (FM) and culture on LJ and MGIT media.

At month 4, when the patient’s weight reached > 40 kg, the moxifloxacin dose was increased to the standard 400 mg/day in accordance with the National Tuberculosis control Program guideline. Unlike most MDR-TB patients on the STR in Rwanda, among whom culture conversion is usually achieved at the second or third month of treatment, this patient remained smear and culture positive at month 4. Hence, kanamycin was continued for another 2 months (up to the maximum of 6 months). Despite the patient’s general improved condition (no fever, no cough, and weight increase to 47 kg at month 10), she remained smear and culture positive, whereas FDA microscopy smears were negative between months 2 and 6, yet turned positive again 1 month after stopping kanamycin (Table 1).

**Table 1:** Treatment and laboratory diagnosis/follow-up of the patient. This table appears in color at www.ajtmh.org.

	M0	M1	M2	M3	M4	M5	M6	M7	M8	M9	M10	M11	M12	M13	M14	M15	M16	M17-M24	M25	M26-M27	M28	M29
Patient’s weight (kg)	37.4	36.7	38.4	40	42	42	44	45	46	46	47	47	47	47.3	47.5	48.5	49	49	50	50	44	43
Xpert MTB/RIF	R																					
FM	3+	3+	2+	17AFB	neg	15AFB	10AFB	12AFB	2+	2+	3+	neg	3+	1+	10AFB	1+	5AFB	neg	1+	neg	neg	neg
FDA	11AFB	1+	Neg	Neg	neg	neg	neg	7AFB	1+	1+	3+	neg	2+	1+	5AFB	neg	1+	neg	neg	neg	neg	neg
Culture MGIT (time to positivity)				pos (20 days)											pos (unknown)					
Culture LJ colony count	3+	1+	4 col	3 col		4 col	10 col	2+	2+	3+	2+	3+	3+	2+	15 col		neg	Neg	cont	neg	cont	NTM
Deeplex-MycTB (genotype)	L 4.6.1					L 4.6.1			L 4.6.1	L 4.6.1	L 4.6.1			L 4.6.1	L 4.6.1							
Isoniazid (mg/day)		**300**			**450**							**600**										
pDST	R					R		R	R	R	R			R	R							
Deeplex-MycTB	R^high^					Rhigh			Rhigh	Rhigh	Rhigh			Rhigh	Rhigh							
WGS	R^high^																					
Pyrazinamide (mg/day)		**800**			**1,200**																	
Deeplex-MycTB	R					R			R	R	R			R	R							
WGS	R																					
Ethambutol (mg/day)		**600**			**800**																	
pDST	R							R	R		R											
Deeplex-MycTB	S					S			S	S	S			S	S							
WGS	R																					
Prothionamide (mg/day)		**500**																				
PDST	R										R											
Deeplex-MycTB	R					R			R	R	R			R	R							
WGS	R																					
Moxifloxacin (mg/day)		*200*			*400*																	
PDST	S					S		S	S	R	R			R	R							
Deeplex-MycTB	S					S			S	R (57% mutant)	R			R	R							
MTBDR-sl LPA	S					S		S	S	R	R											
WGS	S																					
Kanamycin (mg/day)		500			750																	
PDST	S					S		S	S	S	S		S	S								
Deeplex-MycTB	S					S			S	S	S		S	S								
MTBDR-sl LPA	S					S		S	S	R	R											
WGS	S																					
Clofazimine (mg/day)		100										200		100								
Deeplex-MycTB	S					S			S	S	S		S	S								
WGS	S																					
Delamanid												100 mg 2×/day										
Deeplex-MycTB																						
WGS																						
Bedaquiline												400 mg/day	200 mg × 3 per week									
Deeplex-MycTB	S					S			S	S	S			S	S							
WGS	S																					
Linezolid (mg/day)												600 mg/day										
Deeplex-MycTB	S					S			S	S	S			S	S							
WGS	S																					

AFB = acid-fast bacilli; FDA = fluorescein diacetate; FM = fluorescence microscopy; M = month; MGIT = mycobacterial growth indicator tube; NTM = nontuberculous *Mycobacterium*; neg = negative; pDST = phenotypic drug susceptibility testing; pos = positive; R = resistant; S = sensitive; WGS = whole genome sequencing; colony count coding on the LJ (Löwenstein–Jensen) media: < 50 colonies = exact number is given, 1+ = 50–99 colonies, 2+ = 100–199 colonies, and 3+ = 200–499 colonies; color coding for given drug dose: bold = cannot overcome documented resistance, underline = likely effective, and italic = suboptimal increasing the risk of acquired resistance; testing beyond routine includes whole genome sequencing, Deeplex-MycTB, and the monthly phenotypic DST and MTBDR-sl LPA, except for M0, M7, and M10.

As part of routine follow-up testing, samples at months 7 and 8 showed no resistance to second-line drugs. At month 10, FQ resistance was detected by second-line LPA and confirmed by pDST. Repeat second-line LPA and pDST on stored samples and cultures of months 7 and 8 confirmed susceptibility to FQs and second-line injectables, whereas both second-line LPA and pDST revealed FQ resistance on the stored sample and culture of months 9 and 10 (Table 1). At month 11, the patient was changed to bedaquiline (400 mg/day for 1 month and 200 mg × 3 per week after 1 month), linezolid (600 mg), and delamanid (100 mg × 2/day), while continuing with clofazimine (200 mg), isoniazid (600 mg), and pyrazinamide (1,200 mg). Of note, clinicians were unaware of high-level isoniazid and pyrazinamide resistance as first-line LPA or MGIT-PZA was not done, and sequencing was done à posteriori on stored samples. Despite this change of regimen, the patient’s sputum remained smear and culture positive for 5 more months, after which the culture converted to negative from month 16 onward (Table 1).

Consistent with LPA and pDST results, Deeplex-MycTB showed FQ heteroresistance in the sample (57% *gyrA* D94G mutant) and culture isolate (63% D94G mutant) of month 9, yet fully wild-type *gyrA* profiles in previous samples and isolates, in which the bacterial burden was already increasing. Moreover, Deeplex-MycTB revealed high-level isoniazid resistance associated with combined mutations in the *katG* gene (S315T) and *inhA* promoter region (*fabG1* T-8C), as well as pyrazinamide (*pncA* Q10R) resistance in all baseline and follow-up samples and isolates, along with rifampicin (*rpoB* S450L) and ethionamide resistance (*ethA* del GTAATTCAAC and *inhA* promoter mutation, *fabG1* T-8C) previously detected by pDST. The strain phylogenetic profile remained unchanged throughout. No mutation was detected in the *rv0678* or *rrl* targets, associated with resistance to bedaquiline and clofazimine, and linezolid, respectively.

## DISCUSSION

To the best of our knowledge, this is the first study using deep sequencing alongside second-line LPA and pDST to investigate at which timepoint FQ resistance became identifiable in an MDR-TB patient treated with the STR. Despite the ability of Deeplex-MycTB to detect as little as 3% mutant DNA, heteroresistance to FQ was identified in the same month that pDST and second-line LPA detected FQ resistance. Deeplex-MycTB also provided data on mutations in other target genes.

The routinely used second-line LPA and pDST identified FQ resistance at month 10, when the *gyrA* mutation had become fixed (i.e., at least 95% mutant DNA). Both second-line LPA and pDST also detected FQ resistance on a stored sample/isolate, yielding a *gyrA* mutant subpopulation at month 9, confirming their good detection limit of heteroresistance.^8^ Of note, no culture bias (decreased proportion of mutant bacilli in culture relative to direct testing on sample) was observed, suggesting no substantial fitness cost associated with D94G.

A decade of evidence shows good to excellent performance of the STR, with more than 80% success rate in different settings including Rwanda.^3,6^ Although the original Bangladesh STR used gatifloxacin as the core drug, nonavailability of this drug urged programs to use moxifloxacin or levofloxacin. However, recent evaluations revealed superior bacteriological effectiveness of the gatifloxacin-based regimen over levofloxacin- or moxifloxacin-based regimens.^9^ Moreover, the rate of FQ resistance amplification increased from none with gatifloxacin high dose (800 mg/day) to 9.9/1,000 and 14–20.3/1,000, with very high–dose levofloxacin and standard 400 mg/day or high (800 mg) dose moxifloxacin, respectively,^10^ confirming findings from in vitro and a pharmacodynamic infection model.^11,12^ The highest rate of FQ resistance acquisition was recorded for the Stream randomized clinical trial, even with using 800 mg/day moxifloxacin.^13^ Unfortunately, gatifloxacin is currently unavailable. The manufacturer withdrew the drug from the market following a study that showed that the use of gatifloxacin was associated with dysglycemia among elderly treated for pneumonia.^14^ Our previous experience with gatifloxacin showed that dysglycemia was rare in MDR-TB patients treated with the STR, and, when it occurred, it was manageable.^15–17^

Our patient received the low, weight-adapted dose of 200 mg moxifloxacin per day during the most critical first months. Despite clinical improvement there was no bacteriological clearance for 10 months. Retrospective molecular analysis revealed pyrazinamide, prothionamide and high-level isoniazid resistance in the baseline isolate. In combination with the observed phenotypic ethambutol resistance, protection against acquiring resistance to the core drug mainly depended on the companion drugs kanamycin and clofazimine.^1^ When kanamycin was stopped at 6 months, clofazimine was the only likely active companion drug. This led to a growing bacterial population – as evidenced by the increased FM and FDA positivity from month 7 and 8 onwards respectively. Fluoroquinolone resistance became apparent 3 months after stopping kanamycin, and 5 months after switching to standard 400 mg/day moxifloxacin. Surprisingly, several months before any mutants were detectable the bacterial population increased while undergoing FQ drug pressure. The high FQ-minimal inhibitory concentration values conferred by the D94G mutation^12^ cannot be overcome, even by a high dose.

The use of bedaquiline in patients who have been treated with clofazimine is questioned because of possible efflux-pump mediated cross-resistance due to mutations in the *rv0678* gene.^18^ Fortunately, and despite the use of clofazimine as the only likely effective companion drug during 6 months, our patient responded well on the bedaquiline-based regimen and was declared cured after 21 months of bedaquiline-based treatment. No mutation was acquired in *rv0678*.

We documented the appearance of FQ resistance in an STR patient initially receiving a low dose of moxifloxacin, shortly after stopping kanamycin. Despite clinical improvement, bacterial clearance was not obtained and FQ resistance developed. FQ resistance was detected in the same month by Deeplex-MycTB as routinely used second-line LPA and pDST. Increasing counts of acid fast bacilli on FM and FDA microscopy were suggestive of treatment failure. Our patient’s findings emphasize the importance of using high-dose FQ, preferably gatifloxacin, the strongest FQ against TB and the importance of extended molecular and/or pDST at baseline to ensure protection of the core drug effectiveness.
